# Do you hear what I see? Vocalization relative to visual detection rates of Hawaiian hoary bats (*Lasiurus cinereus semotus*)

**DOI:** 10.1002/ece3.3196

**Published:** 2017-07-20

**Authors:** Paulo. Marcos Gorresen, Paul M. Cryan, Kristina Montoya‐Aiona, Frank J. Bonaccorso

**Affiliations:** ^1^ Hawai'i Cooperative Studies Unit University of Hawai'i at Hilo Hilo HI USA; ^2^ Fort Collins Science Center U.S. Geological Survey (USGS) Fort Collins CO USA; ^3^ Pacific Island Ecosystems Research Center USGS Hawaii National Park HI USA

**Keywords:** acoustic detection, behavior, echolocation, non‐vocalization, thermal infrared video, vocalization

## Abstract

Bats vocalize during flight as part of the sensory modality called echolocation, but very little is known about whether flying bats consistently call. Occasional vocal silence during flight when bats approach prey or conspecifics has been documented for relatively few species and situations. Bats flying alone in clutter‐free airspace are not known to forgo vocalization, yet prior observations suggested possible silent behavior in certain, unexpected situations. Determining when, why, and where silent behavior occurs in bats will help evaluate major assumptions of a primary monitoring method for bats used in ecological research, management, and conservation. In this study, we recorded flight activity of Hawaiian hoary bats (*Lasiurus cinereus semotus*) under seminatural conditions using both thermal video cameras and acoustic detectors. Simultaneous video and audio recordings from 20 nights of observation at 10 sites were analyzed for correspondence between detection methods, with a focus on video observations in three distance categories for which accompanying vocalizations were detected. Comparison of video and audio detections revealed that a high proportion of Hawaiian hoary bats “seen” on video were not simultaneously “heard.” On average, only about one in three visual detections within a night had an accompanying call detection, but this varied greatly among nights. Bats flying on curved flight paths and individuals nearer the cameras were more likely to be detected by both methods. Feeding and social calls were detected, but no clear pattern emerged from the small number of observations involving closely interacting bats. These results may indicate that flying Hawaiian hoary bats often forgo echolocation, or do not always vocalize in a way that is detectable with common sampling and monitoring methods. Possible reasons for the low correspondence between visual and acoustic detections range from methodological to biological and include a number of biases associated with the propagation and detection of sound, cryptic foraging strategies, or conspecific presence. Silent flight behavior may be more prevalent in echolocating bats than previously appreciated, has profound implications for ecological research, and deserves further characterization and study.

## 1 INTRODUCTION

All bats in the suborder Yangochiroptera (formerly Microchiroptera) echolocate (Altringham, 2011), yet for most of these species, the continuity of vocalization during flight remains underexplored. Determining whether and when bats may forgo vocalizing as part of echolocation is important for our general understanding of sensory ecology (Geva‐Sagiv, Las, Yovel, & Ulanovsky, 2015), and particularly for validating the underlying assumptions of scientific research, management, and conservation methods that rely on acoustic detection of echolocation calls as indices of bat presence, activity levels, and habitat use (e.g., Loeb et al., 2015; Walters et al., 2012).

A primary function of bat echolocation is to detect and localize prey at near distances (<20 m; Griffin, 1958; Holderied & von Helversen, 2003). However, known foraging strategies of insectivorous bats include sensory systems that do not require active sound production (Altringham & Fenton, 2003). Bats that glean from surfaces can passively listen for sounds produced by prey, briefly stop calling or reduce call intensity just before prey capture or landing (Faure & Barclay, 1994), and make use of visual cues when searching for food (Eklöf & Jones, 2003). Interrupting or inconspicuously using echolocation may benefit bats in more ways than stealthily approaching prey. In both foraging and social contexts, bats are known to change their echolocation calls or stop echolocating when other bats (including individuals of different species) are nearby, possibly sometimes for cooperative reasons (Chiu & Moss, 2008; Chiu, Xian, & Moss, 2008; Li et al., 2014); reasons for this type of “silent behavior” remain uncertain.

In addition to biological reasons that bats might not actively echolocate, the acoustic detectability of bat calls is known to vary with environmental conditions and sampling equipment (Adams, Jantzen, Hamilton, & Fenton, 2012; Stilz & Schnitzler, 2012). Therefore, studying the underlying biology associated with bats potentially forgoing vocalization can be complicated by the methodological challenges of consistently detecting a given species' calls. Knowing when a species stops vocalizing for biological reasons is as important as knowing when the calls of a species are less detectable, because both have profound implications for acoustic‐based monitoring efforts.

Early studies that paired thermal videography with acoustic sampling at wind turbines first alerted us to the possibility that rates of bat vocalization might be low relative to the occurrence of individuals detected visually. In a 3‐month study at a wind power facility in Indiana, Cryan et al. (2014) noted that only 49% of video detections of bats flying near acoustic recorders mounted atop turbines had any associated acoustic detections, suggesting that bats might sometimes forgo sound production while flying close to wind turbines. In a similar 6‐month study, concurrent acoustic and video sampling of the airspace immediately around turbines resulted in echolocation recordings of Hawaiian hoary bats (*Lasiurus cinereus semotus*) on only 8% of the nights sampled, whereas bats were detected by video on 86% of the same nights (Gorresen, Cryan, Huso, et al., 2015). These results indicated that bats in flight might at times be less vocal than assumed, at least near wind turbines and perhaps other tall, emergent structures. However, comparable studies that paired video and acoustic sampling for this or other echolocating species in more natural environments are lacking, so it is unknown whether the behaviors of bats at wind turbines are typical or related in some way to those unnatural situations. Additional evidence that hoary bats in particular may sometimes forgo calling during flight comes from repeated observations of individuals that were not acoustically detected prior to contacting mist nets, while circling conspecifics captured in mist nets, and during release following capture (Weller & Giordano, 2013). In each of the latter cases, nearby acoustic detectors regularly failed to record echolocation calls.

The objective of this study was to determine whether Hawaiian hoary bats seen flying at close range always produced detectable echolocation calls and if not, to elucidate possible biological and methodological reasons underlying the absence of acoustic detections. We examined flight behavior and acoustic characteristics to determine whether bats seen but not heard stopped vocalizing for brief periods or engaged in behaviors that may have resulted in us not detecting them acoustically. We assessed how linearity of flight paths, the proximity of bats to cameras and acoustic detectors, and the number of individuals present affected the frequency of detections. Finally, we examined vocalization characteristics that may have affected detectability.

## 2 METHODS

This field study was conducted from 8 September 2014 through 14 October 2014, approximately 9 miles south of Hilo, Hawai'i Island, USA (19°36′40″ N, 155°05′00″ W). At an elevation of about 230 m, vegetation at the site consists of a checkerboard‐like orchard of short (approx. 5 m) macadamia trees (*Macadamia integrifolia*) bordered by tall (approx. 40 m) windbreaks of Cook's pine (*Araucaria columnaris*) along a grid of roadways. The resulting airspace available to foraging or commuting bats was a mix of edge‐space and open‐space settings above the orchard. Twenty nights of time‐synchronized video and acoustic samples were collected as part of a study of bat response to dim ultraviolet illumination (Gorresen, Cryan, Dalton, et al., 2015). The dataset analyzed herein was derived from the “control” sample of nights, that is, the subset of nights at 10 orchard sites prior to application of ultraviolet illumination. Weather conditions during all but one night were suitable for effective acoustic and video sampling (low to no wind, moderate temperatures and humidity, and little to no rain; nightly metrics are detailed in Table S1).

### 2.1 Acoustic sampling and metrics

Bat echolocation was monitored with ultrasonic detectors (Song Meter 2 Bat+, Wildlife Acoustics, Concord, MA), with microphones set at a 2 m height and oriented at a 45° angle to the ground toward the airspace imaged by video cameras. The omnidirectional microphone of the detector (SMX‐II, Wildlife Acoustics) had a 360° beam pattern (Andrews, Staton, & Latham, 2011), thus sampling a larger volume of airspace than the video cameras (see below). Detectors were programmed to begin recording 30 min before local sunset until 30 min after sunrise the next morning. Detector clocks were checked and synchronized with video camera clocks using the same computer on a weekly basis. Detector and video cameras time signals never varied by more than a few seconds between weekly checks. Acoustic events were recorded without digital compression, as full‐spectrum wav sound files with the following settings: sampling rate of 192 kHz; high‐pass filter at 1,000 Hz and 36 decibel (dB) gain; microphone bias off; digital high‐pass filter at fs/24; digital low‐pass filter off; trigger level 18 signal‐noise ratio; trigger window 2.0 s; trigger max length 15 s; frequency division ratio 16.

We used the program CallViewer (version 18; Skowronski & Fenton, 2008) to identify and characterize bat call signals (i.e., pulses). All files (inclusive of those classed by algorithms as “noise”) were visually inspected as spectrograms to ensure that designations did not include false positives (files misidentified as containing bat calls) or false negatives (files with missed bat calls). Spectrograms of bat calls were used to visually relate specific pulses with metrics of call structure, to identify unusual vocalizations indicative of social calls (Pfalzer & Kusch, 2003), and to infer feeding activity by the occurrence of terminal‐phase calls (“feeding buzzes”; Griffin, 1958; but see Russo, Ancillotto, Cistrone, & Korine, 2015). Terminal‐phase calls were qualitatively distinguished from search‐phase and approach‐phase calls by a rapid increase in the call rate. The search‐phase portions of calls were identified as a successive series of pulses separated by at least 100 ms (sensu Barclay, Fullard, & Jacobs, 1999).

CallViewer produced output from which we extracted the following metrics of call structure: pulse duration (ms), minimum and maximum frequency, and frequency of maximum energy (kHz). The metrics were subsequently summarized for a randomly selected subset of 109 recordings that included the search‐phase portions of calls, given that they contained a series of ≥3 consecutive pulses ≥15 dB at the frequency of maximum energy (Fig. S1). For each pulse, we calculated bandwidth (difference in kHz between the minimum and maximum frequency) and modulation (percent difference in bandwidth relative to maximum frequency). For the series of pulses comprising the search‐phase portion of the call, we calculated the interpulse interval (IPI; difference in millisecond between the onset of successive pulses; sensu Petrites, Eng, Mowlds, Simmons, & Delong, 2009) and pulse rate (number of signals per second, or Hz; difference in millisecond between the onset of the first and end of the last of a series of successive pulses, divided by number of pulses).

### 2.2 Visual sampling and metrics

Bat occurrence and behavior were monitored using thermal surveillance cameras (Axis Q1922‐E, Axis Communications, Lund, Sweden) that imaged an area of the far infrared spectrum (9,000 − 14,000 nm) and required no supplemental illumination. At each sample site, a surveillance camera was set alongside the windbreak and aimed toward the airspace over the macadamia trees. Each camera was equipped with a 10 mm lens, had a horizontal viewing angle of 57°, and the 640 by 480 pixel sensor imaged a field of view that was 54 m wide by 41 m high at a distance of 50 m, broadening further with distance. Based on sensor resolution and lens focal length, we estimate that our cameras detected flying bats out to a maximum distance of about 80 m from the camera. Considering the three‐dimensional volumes of airspace sampled by the cameras and detector microphones out to an arbitrarily chosen distance of 50 m, we estimate that our surveillance cameras imaged about 36,900 m^3^, or approximately 14% of the estimated 261,799 m^3^ of airspace sampled at that distance by the omnidirectional microphones of the acoustic detectors.

Video imagery was processed using custom‐written code (Matlab R2013b with Image Processing Toolbox, Mathworks Inc., Natick, Massachusetts, USA) to automatically detect animals flying through the video scenes (code is available as the supplementary material in Cryan et al., 2014 and Gorresen, Cryan, Huso, et al., 2015). Video was recorded at 30 frames per second, and every 10th video frame was analyzed, resulting in the detection of events of at least 0.3 s duration. All objects detected by software algorithms were visually reviewed and characterized as to identity, proximity, and flight behavior. Given the size of the field of view (FOV) and expected flight speed of hoary bats (≤7 m/s; De La Cueva Salcedo, Fenton, Hickey, & Blake, 1995), few bats could have been missed within the FOV at the sampling rate we used. Video processing entailed use of a size filter that eliminated very small targets (<5 contiguous pixels). This size filtering effectively reduced the number of nuisance detections of insects at relatively close range and excluded bats outside the range of consistent video detection at distances greater than about 80 m. One shortcoming of thermal imagery is the lack of visual cues for perceiving the depth of an object within a scene, typically provided by shadows in reflected‐light imagery. We discriminated distant bats from nearby insects in the thermal imagery using a combination of the speed at which they flew through the scene and the sharpness of the image borders. Bats flying close to and far from the cameras, almost always had sharp image borders and/or clear bat‐like shapes, and transited scenes quickly or slowly, respectively. Insects imaged by the cameras almost always had blurry borders, indistinct shapes, and usually transited scenes quickly.

Nightly measures of bat activity derived from video consisted of the total number of detections and proximity. Proximity was assessed based on the nearest approach by a bat to the camera, and classed as either a near‐range (≤25 m), mid‐range (>25 to 50 m), or far‐range detection (>50 m, but to a maximum of 80 m) (Fig. S2). Proximity assessment from video was calibrated using targets of known size at known distances. Bats detected at a distance of ≤50 m were identified by their characteristic body shape and flight, whereas bat detected at distance >50 m were generally identified solely by flight pattern and may be under‐represented in the resulting tallies of bat occurrence. Flight behavior was qualitatively designated as straight, curved, or erratic based on whether the flight path was linear or included one or more than one curves or loops during the duration of the detection. In cases where two or more bats were concurrently visible, behavior was recorded as agonistic when individuals flew within a few meters of each other and interacted with sharp turns and chases. Foraging or transiting flight by two or more bats that did not appear to follow one another or change trajectory was recorded as noninteracting.

Visual detections were matched with bat calls by examining corresponding time stamps for each set of recordings. Any call file that occurred within 30 s of the video detection was considered a potential match. The median difference in time between acoustic and visual detections was 2 s, indicating little potential for mismatches. We tested for homogeneity among proximity, flight type, and acoustic detection classes using Fisher's exact test and controlled for family‐wise error using Bonferroni correction in R (version 3.1.2; R Core Team, 2014).

## 3 RESULTS

A total of 2,535 acoustic and 784 visual (videographic) detections of bats were recorded over 240 hr during the 20 nights of sampling (Table 1). Overall, the nightly totals of acoustic and visual detections were moderately correlated (*r* = .64, *p *=* *.0024). However, on a nightly basis, an average of only 31% of the visual detections had associated synchronous (matching) bat calls. Three nights had no matching detections (9/18, 9/24, 9/25) and corresponded to samples where there were a moderate number of visual but few acoustic detections. Several nights had a high number of acoustic detections but relatively few visual detections. Four nights had >50% matching detections (9/10, 9/11, 9/12, 10/8), yet high correspondence (95%) between visual and acoustic detections only occurred on a single night (10/8). Nine nights, or nearly half of those we sampled, had relatively high numbers of visual detections but little or no acoustic activity, clearly demonstrating that flying bats detected by video were often not detected acoustically. A weak correlation was noted between acoustic activity and moon phase (*r* = .47, *p *=* *.0364), but not between video detections and moon phase (*r* = .28, *p *=* *.2269), indicating that bats were prevalent in both dark and bright periods (new on 9/24 and full phases on 9/9 and 10/8).

**Table 1:** Number of acoustic and visual events by night. Proportion refers to the frequency of synchronous acoustic–visual detections relative to the total number of nightly visual detections

Date	Acoustic	Visual	Synchronous	Proportion (%)
09/08	19	24	12	50
09/09	24	43	13	30
09/10	324	20	13	65
09/11	291	7	4	57
09/12	79	3	2	67
09/15	36	19	3	16
09/16	229	47	21	45
09/17	330	27	9	33
09/18	2	56	0	0
09/19	7	24	1	4
09/22	7	13	1	8
09/23	3	17	1	6
09/24	3	22	0	0
09/25	1	20	0	0
10/06	30	15	5	33
10/07	74	16	3	19
10/08	496	243	232	95
10/09	347	107	49	46
10/13	157	32	10	31
10/14	76	29	4	14
Total	2,535	784	383	
Average	127	39	19	31

Synchronous visual and acoustic detections occurred more frequently for near‐range than for mid‐range to far‐range detections (Table 2; *p *<* *.0001). However, although near‐range (≤25 m distance) detections comprised 55% (*n *=* *434) of all visual observations, acoustics were not recorded for as much as 35% of this subset of detections. The proportion of visual detections lacking acoustics was even greater (75%) at mid‐range distances (>25 to 50 m). Far‐range (≥50 m) detections made up only 5% (*n *=* *41) of all visual detections, yet acoustics were recorded in about half of those cases (56%).

**Table 2:** Number and proportion of visual detections with and without associated acoustic detections relative to proximity and the type of flight observed by video

Proximity	Flight type	Acoustics	No acoustics	Subtotal
Near (≤25 m)	Straight	153 (57%)	114 (43%)	267 (62%)
	Curved	116 (80%)	29 (20%)	145 (33%)
	Erratic	15 (68%)	7 (32%)	22 (5%)
	Subtotal	284 (65%)	150 (35%)	434 (55%)
Mid (>25–50 m)	Straight	22 (15%)	122 (85%)	144 (47%)
	Curved	45 (37%)	78 (63%)	123 (40%)
	Erratic	9 (21%)	33 (79%)	42 (14%)
	Subtotal	76 (25%)	233 (75%)	309 (39%)
Far (>50 m)	Straight	5 (100%)	0 (0%)	5 (12%)
	Curved	11 (50%)	11 (50%)	22 (54%)
	Erratic	7 (50%)	7 (50%)	14 (34%)
	Subtotal	23 (56%)	18 (44%)	41 (5%)
	Total	383 (49%)	401 (51%)	784

Hawaiian hoary bats often repetitively flew close (2–3 m) to trees and the ground along unpaved roads separating the macadamia and pine trees, although most were observed in the open airspace above the orchard. Most visual observations of bats involved individuals moving along straight (53%; *n *=* *416) and curved (37%; *n *=* *290) flight paths. A smaller proportion (10%; *n *=* *78) involved erratic flight suggestive of foraging and close pursuit of prey. Among near‐range and mid‐range detections, there were proportionally fewer straight and more curved flight paths associated with acoustics than expected (*p *<* *.0001 and *p *=* *.0011, respectively). The occurrence of acoustics during far‐range detections was not significantly related to flight path type (*p *=* *.4692).

Of the 2,535 distinct call file recordings, about 10% (*n *=* *250) included terminal‐phase calls and demonstrated active foraging in the study area. Another 2% (*n *=* *42) of call recordings were categorized as “social.” Bats emitted search‐phase calls that ranged from a series of relatively long, shallow‐modulated pulses to short, steep‐modulated pulses (Table 3; Fig. S3), sometimes exhibiting considerable structural variability within the same call sequence (Fig. S4). Echolocation pulses were centered at a mean peak frequency of 29.3 kHz with a descending modulation of about 26% from a mean of 35.6–26.3 kHz. The interpulse interval (IPI) of calls averaged 217 ms (±5 *SE*) and pulse rates averaged 6.7 Hz (±0.1 *SE*). However, pulse rates were sometimes considerably lower, with minimum and 1st quartile values of 2.6 and 4.9 Hz, respectively, and with correspondingly long IPI values of 271 (3rd quartile) and 680 ms (maximum). The call structure recorded from bats observed in chases, or those identified as social calls were often comprised of overlapping shallow‐modulated and steep‐modulated pulses, with many pulses at a lower peak frequency (<20 kHz) and longer duration than those typical of search‐phase portions of calls (Figs S5 and S6).

**Table 3:** Description of the search‐phase components of calls by *Lasiurus cinereus semotus*. Characteristics include interpulse interval (IPI), pulse rate and duration, fundamental start, peak, and end frequencies, bandwidth, and modulation (% sweep of start frequency). A total of 584 pulses were characterized in the 109 call files examined

	IPI (ms)	Rate (#/s)	Duration (ms)	Start freq. (kHz)	Peak freq. (kHz)	End freq. (kHz)	Bandwidth (kHz)	Modulation (%)
Mean	217	6.7	6.9	35.6	29.3	26.3	10.1	25.7
*SD*	98	2.3	3.5	8.5	5.0	3.7	6.7	11.0
*SE*	5	0.1	0.2	0.4	0.2	0.2	0.3	0.5
Min	81	2.6	1	23.3	21.8	11.3	0.8	2.8
1st qrt	140	4.9	4	29.3	25.5	23.3	5.3	18.4
Median	191	6.7	7	33.4	28.5	26.3	8.3	24.3
3rd qrt	271	8.4	9	39.0	31.5	28.5	12.0	31.8
Max	680	12.3	21	64.5	55.5	44.3	38.3	69.9

Multiple bats were visible in only 3% of the 784 visual samples, with two bats (*n *=* *26) or three bats (*n *=* *1) comprising these events. There were five instances in which bats engaged in close and sustained chasing behavior (aerial “dogfight”), but acoustics were not detected in three of those interactions even though two occurred at mid‐range. Most observations (67%, *n *=* *18) of multiple bats for which no acoustics were recorded involved individuals flying past each other, with no apparent change in flight trajectories or interactions (Table 4).

**Table 4:** Number and proportion of visual detections with and without associated acoustic detections relative to proximity and agonistic interactions observed by video

Proximity	Interaction	Acoustics	No acoustics	Subtotal
Near (≤25 m)	Yes	0 (0%)	0 (0%)	0 (0%)
	No	0 (0%)	6 (100%)	6 (100%)
	Subtotal	0 (0%)	6 (100%)	6 (22%)
Mid (>25–50 m)	Yes	1 (33%)	2 (67%)	3 (18%)
	No	3 (21%)	11 (79%)	14 (82%)
	Subtotal	4 (24%)	13 (76%)	17 (63%)
Far (>50 m)	Yes	1 (50%)	1 (50%)	2 (50%)
	No	1 (50%)	1 (50%)	2 (50%)
	Subtotal	2 (50%)	2 (50%)	4 (15%)
	Total	6 (22%)	21 (78%)	27

## 4 DISCUSSION

Our simultaneous video and audio recordings of Hawaiian hoary bats flying under seminatural conditions revealed the unexpected outcome that bats often flew near acoustic monitoring devices without emitting detectable vocalizations. Echolocation calls were detected, on average, only about a third of the time that we visually observed bats with thermal cameras. There are a number of possible reasons why we often “saw” Hawaiian hoary bats without “hearing” them. Such reasons might not be mutually exclusive and could range from various sampling biases to particulars of bat ecology and behavior. An often unstated assumption of studies involving nongleaning bats is that most species capable of echolocation will consistently call during flight, and silent behaviors in aerial hawking species were only recently discovered (Chiu & Moss, 2008; Chiu et al., 2008). Exploration of potential exceptions to one of the most widespread assumptions about bat echolocation has the potential to uncover new understanding for designing studies and interpreting acoustic data.

### 4.1 It's not them, it's us

Some of the mismatch we observed between visual and acoustic detections may have been attributable to the difficulty of sampling three‐dimensional airspace with video cameras and acoustic recorders. As expected, matches between acoustic and visual detections occurred proportionally more for near‐range than for mid‐range to far‐range detections, likely indicating that nearby bats were more readily detected acoustically than those flying at greater distances (Skowronski & Fenton, 2009). Nevertheless, although near‐range detections comprised over half of all visual observations, as many as a third of those video detections lacked accompanying acoustics. The proportion of visual detections lacking acoustics was even greater at mid‐range distances. Interestingly, although far‐range detections made up only a very small proportion of all visual detections, bat calls were detected in about half of those cases. Although our results indicate that distance factored into our ability to acoustically detect Hawaiian hoary bats, other sampling biases also likely existed.

Detection of ultrasound depends in part on the angle between a sound source and detector (Adams et al., 2012). Echolocation calls of bats can be highly directional (Hulgard, Moss, Jakobsen, & Surlykke, 2015), and aerial hawking bats can increase directionality to extend sonar range (Jakobsen, Brinkløv, & Surlykke, 2013). In addition, some species are capable of aiming echolocation “beams” off the direction of flight (Fujioka et al., 2014) and alternating the direction of those beams between successive pulses (Seibert, Koblitz, Denzinger, & Schnitzler, 2013). Although vocalization by *L. c. semotus* can include low‐frequency pulses that are readily detected at a distance, those directed off the axis of the line of flight will be harder to detect than forward‐pointing calls by a bat orientated toward an acoustic detector. Consequently, a fraction of calls might go undetected even when bats are relatively close to an acoustic detector.

In addition to the biasing effects of distance and angle, bat flight patterns likely influenced detectability. Within the field of view imaged by thermal cameras, most visual detections involved bats flying on straight or curved paths. Only about 10% involved erratic flight paths indicating close pursuit of prey. The detection of bat calls was significantly under‐represented during straight flight at near‐range and mid‐range distances. In contrast, acoustics were more frequently associated with bats flying a curved path. As discussed above, bats flying straight and emitting highly directional calls might be harder detect from a given monitoring location because their calls only point along one axis, particularly in the airspace close to their bodies. Alternatively, our results suggest that Hawaiian hoary bats might vocalize at lower rates when engaged in linear flight, resulting in longer interpulse intervals than bats flying more circuitous paths, particularly if the latter activity involves searching for prey or probing the background auditory scene. Moreover, commuting is generally faster than foraging flight speeds (Grodzinski, Spiegel, Korine, & Holderied, 2009), and observations of Hawaiian hoary bats flying straight paths may partly consist of individuals calling less frequently or forgoing echolocation while transiting to and from regularly used foraging patches.

Variability in the structure of hoary bat calls may partially explain lack of correspondence between video and acoustic detections. The search‐phase echolocation calls of *L. c. semotus* that we recorded included both the long‐duration, narrowband (shallow) and short‐duration, broadband (steep) types (Fig. S3). The former are generally used by bats foraging for prey in open airspace, and such calls are optimized for distant detection of weak echoes, whereas short, broadband calls differ in that they tend to be used by bats foraging for prey near acoustic clutter and needing to determine their position in space relative to a background (Schnitzler, Moss, & Denzinger, 2003). Climate conditions may also cause spatial and temporal variability in the call structure of bats, with some bats possibly switching to shallow, lower‐frequency calls when climate conditions are such that sound is highly absorbed and attenuated by the atmosphere (e.g., high humidity; Griffin, 1971; Snell‐Rood, 2012). An individual bat may adjust its call type between shallow and steep search‐phase calls to meet context‐specific foraging needs (Schnitzler & Kalko, 2001; Snell‐Rood, 2012), and hoary bats are no exception (O'Farrell, Corben, & Gannon, 2000; Obrist, 1995). For example, a bat chasing insects within vegetative clutter and using steep calls may change to shallow, lower‐frequency calls as it ascends into open airspace to forage away from obstacles and/or in more humid conditions. Hawaiian hoary bats regularly fly within more cluttered vegetation than *L. c. cinereus* (Barclay, 1985; Jacobs, 1996), and documented calls of *L. c. semotus* fall within the higher‐frequency and shorter‐duration range of variation exhibited by the mainland subspecies (O'Farrell et al., 2000). Hawaiian hoary bats can emit shallow calls (e.g., O'Farrell et al., 2000), presumably to enhance long‐range target detection or aid navigation when foraging away from edge‐space settings. Because the sound of longer duration, shallow calls travels farther through air, such calls would be more detectable with acoustic devices, possibly accounting for the greater proportion of concurrent acoustic and visual detections of bats at longer ranges. As our cameras and bat detectors were on the ground and thus amidst vegetative clutter, bats flying closest to these sensors were more likely to emit shorter‐duration, higher‐frequency calls that could attenuate more quickly in humid air and possibly be harder to detect. However, if variability in the structure of calls was a major source of detection bias, we would predict that bats flying farther above the ground and away from vegetation would be more frequently detected than those flying near our sensors in the clutter, which was not the pattern we observed with our near and mid‐distance detections.

The intervals at which bats emit calls might be another partial explanation for low correspondence between video and acoustic detections. We frequently recorded call sequences with low pulse rates and long interpulse intervals, which suggests long‐range targeting of insect prey by bats in the study area, or possibly the presence of conspecifics. Our observed pulse rates averaged approximately 7 per second, with rates lower than 5 per second in about a quarter of the search‐phase pulses we examined. The previously reported call rate of foraging Hawaiian hoary bats was much higher, averaging about 12 pulses per second (Belwood & Fullard, 1984), a difference that may reflect dissimilar study settings or prey availability. The site studied by Belwood and Fullard (1984) on Kaua'i was surrounded by dense native forest and located among several buildings illuminated by incandescent lights where bats foraged on concentrations of insects; as such, call parameters may have reflected echolocation targeting near‐range prey. In contrast, the relatively sparse insect prey available at our study site (see Gorresen, Cryan, Dalton, et al., 2015) and the open‐space setting above the orchard may have influenced foraging bats to use lower pulse rates, and correspondingly longer interpulse intervals. Wide‐ranging bats challenged with locating sparsely distributed prey sometimes make two or more wing beats without emitting calls and prolong interpulse intervals to expand search range (Holderied & von Helversen, 2003; Schnitzler et al., 2003). The presence of conspecifics can also lead to lower pulse rates and longer interpulse intervals in *L. c. cinereus* and other bat species (Cvikel et al., 2015; Obrist, 1995) although our observations of conspecific interactions were fewer than those reported by Belwood and Fullard (1984), who observed groups of up to eight individuals. Regardless of reasons for the low pulse rates we observed, it might be harder to detect the relatively few pulses of a bat emitting long interpulse intervals as it flies rapidly through the small volume of airspace sampled by an acoustic detector. On the other hand, given our observed mean pulse rate, even a short flight segment taken through the sample volume by a bat flying at high speed (e.g., averaging 11.1 m/s in open airspace by foraging *L. c. semotus*; Belwood & Fullard, 1984) would still likely result in the detection of several pulses if it were vocalizing.

The atmospheric attenuation of sound is strongly dependent on humidity, which limits the effective range of bat echolocation, particularly for bat calls emitted at high frequencies (Griffin, 1971; Lawrence & Simmons, 1982; Snell‐Rood, 2012). Echolocation of large landscape objects such as forest edges may entail an acoustic perception range of no more than 100 m (Holderied & von Helversen, 2003) and perhaps <50 m (Stilz & Schnitzler, 2012). However, the unidirectional transmission of sound between a source and detector may be expected to be greater than the range of attenuated echoes perceived by a bat. Moreover, the frequency range of calls recorded at our study site was relatively low (mean = 29.3 kHz; 95th percentile of peak frequency = 38.1 kHz). Given that Hawaiian hoary bats were often detected acoustically at distances ≥50 m, atmospheric attenuation may only explain some of the missed acoustic detections despite the relatively high humidity (mean nightly maximum: 87%) at our study site.

### 4.2 It's not us, it's them

Overall, explanations involving distance, angle, flight path, call rate, call structure, and atmospheric attenuation all still fall short in providing a conclusive explanation for the frequent absence of calls detected from bats at near‐range. Foremost among possible biological explanations for why Hawaiian hoary bats may forgo vocalization during flight is that they may not always need to echolocate. Bats integrate signals from multiple senses, in certain situations showing evidence of relying more heavily on cues other than sound (Suthers, 1970; Thomas, Moss, & Vater, 2003). Bats in open‐air flight are seldom in complete darkness, and there is usually some light available to them at night (Davis & Barbour, 1965; Martin, 1990), and several species have been shown to use vision for long‐range orientation, navigation, and the avoidance of large obstacles (Chase, 1981; Griffin, 1970; Suthers, 1970; Tsoar et al., 2011; Williams & Williams, 1967). The ability of bats to augment acoustic prey detection with visual information at close range may also be important or preferred, particularly where there is adequate lighting (Eklöf & Jones, 2003; Eklöf, Tranefors, & Vázquez, 2002; Orbach & Fenton, 2010). For example, Barclay et al. (1999) observed Hawaiian hoary bats detecting and initially tracking moths without vocalizing and presumably using vision in bright artificial light; these bats only seemed to vocalize while closing in on a targeted moth. However, a companion effort to the study reported here showed that bat acoustic activity in the macadamia orchard was positively associated with moon illumination, whereas visual detection rates were not (Gorresen, Cryan, Dalton, et al., 2015). That is, although bats observed by video at our site were prevalent in both dark and bright periods of the moon, we detected increases in echolocation activity during well‐lit periods rather than the decreases expected if sight‐over‐sound preference reduced vocalization. We do not know why these bats echolocated more in moonlight, but note that ambient illumination might be another factor complicating acoustic methods if this phenomenon occurs more broadly. Acoustic monitoring efforts rarely account for ambient levels of nighttime illumination as a covariate of bat presence and/or activity.

It is possible that bats echolocate less when moving through familiar landscapes. Hawaiian hoary bats often predictably visit the same foraging areas on consecutive nights (Bonaccorso, Todd, Miles, & Gorresen, 2015), a behavior also noted in other studies of insectivorous bats (e.g., Entwistle, Racey, & Speakman, 1996; Meyer, Weinbeer, & Kalko, 2005; Wai‐Ping & Fenton, 1989). Bats foraging in a familiar natural environment are thought to use “foraging beats” and stereotypic flight paths, as well as rely on an internal map of their surroundings to reduce echo‐processing load and enable greater focus on tracking prey (Hulgard et al., 2015). Similarly, bats negotiating obstacles in a laboratory flight room also reduce call rates as they gain familiarity with their flight space (Barchi, Knowles, & Simmons, 2013; Holland & Waters, 2007). The regularly patterned use of foraging areas by Hawaiian hoary bats and the stereotypic flight of bats often observed by video in this study may indicate individuals flying in familiar spaces that are calling at lower rates and longer intervals, or occasionally forgoing echolocation. Moreover, although the energetic cost of echolocation is thought to be low (Speakman & Racey, 1991), a potential drawback of continuous echolocation in frequently used areas might be the attraction of predators capable of hearing audible components of calls.

Bats sometimes forage for insects within hearing range of each other, and aerial “dogfights” and chases among foraging bats of the same species have been reported in natural settings (Rydell, 1986; Simmons, Eastman, Horowitz, O'Farrell, & Lee, 2001). Lower calling rates in the presence of conspecifics have been observed in *L. c. cinereus* and other species of bats, indicating that vocalization for prey targeting can be influenced by social interactions (Cvikel et al., 2015; Obrist, 1995). Agonistic interactions can intensify among individuals of some insectivorous species when prey is scarce, and social calls may be used to warn off intruding bats and forestall kleptoparasitism (Barlow & Jones, 1997; Belwood & Fullard, 1984; Budenz, Heib, & Kusch, 2009). In some cases, echolocation may be wholly absent, and agonistic calls are the only vocalizations used during interactions (Rydell, 1986). In contrast, a hypothesized cause of the silent behavior observed in close‐flying big brown bats (*Eptesicus fuscus*) is that going silent may be a cooperative hunting strategy conspecifics use to benefit from the echoes of others (Chiu & Moss, 2008; Chiu et al., 2008). Hawaiian hoary bats were seldom observed by video in the same airspace at the same time, and when multiple individuals were seen flying together, most detections involved individuals flying by each other, with no apparent change in flight trajectories or agonistic interactions. In the few cases where interactions were observed visually, most had no associated acoustics. We know too little about the social interactions of *L. cinereus* to speculate on why they might forgo echolocation in the presence of conspecifics if that is indeed what was happening in our study.

The infrequency of recorded interactions among individual bats is notable and may have to do with a strongly structured use of foraging space, as demonstrated in a separate radio tracking study (that included our study site), in which adult male bats showed no overlap in core‐use foraging areas and only little overlap among individuals of other sex and age groups (Bonaccorso et al., 2015). By avoiding spatial overlap, resident bats minimize competition, yet the process is likely dynamic in that bats may routinely update the occupancy status of adjacent foraging areas. That is, Hawaiian hoary bats regularly transit among multiple foraging areas throughout a night (Bonaccorso et al., 2015), and acoustic and video recordings show that the monitored airspace is not continually occupied. The low‐frequency calls emitted by Hawaiian hoary bats may alert foraging individuals to the presence of a new arrival or an encroaching neighbor at a relatively long‐distance. Consequently, interlopers may seek to avoid attracting the attention of bats already present by briefly going quiet during transit.

If Hawaiian hoary bats exhibit silent behavior, it has the potential to be a seasonal phenomenon that may not occur throughout the year. For example, our study took place during a time of year when many female Hawaiian hoary bats are thought to have finished weaning young (Menard, 2001) and during which aggregations of *L. c. semotus* have been observed around Hawai'i Island (Tomich, 1986; Fujioka & Gon, 1988; M. Gorresen, personal observations). Hawaiian hoary bats tend to be solitary and dispersed during other seasons, and the fact that most aggregations in Hawai'i have been observed between August and the end of November indicates seasonal changes in population social structure, possibly driven by reproduction. In North America, many adult and juvenile *L. c. cinereus* of both sexes show signs of mating readiness by September (Cryan et al., 2012; Druecker, 1972), and as in Hawai'i, most observations of hoary bat aggregations on the mainland were made from approximately early September through late October (Cryan & Brown, 2007). Virtually nothing is known about the actual mating habits of hoary bats (Cryan, 2008), particularly whether courtship and mating behaviors involve specialized vocalizations. Although mating calls of multiple species of distantly related insectivorous bats with similar habits to *L. cinereus* have been well characterized in Europe (Pfalzer & Kusch, 2003), we are not aware of any evidence indicating that *L. cinereus* seasonally uses specialized mating calls. Considering our observations of Hawaiian hoary bats in this context, we speculate that silent periods during flight, for whatever reason, may have evolved as a different seasonal strategy to specialized mating vocalizations. It may not be coincidence that other instances we cited above all took place during the late summer and autumn. A broader understanding of the seasonal continuity of vocalization by *L. c. semotus* during flight clearly is needed.

Our observations strongly suggest that at least some of our inability to detect calls from flying Hawaiian hoary bats was attributable to the biological explanation that bats sometimes stop vocalizing during flight. However, this analysis was opportunistic, and our study was not optimally designed to rule out the possibility that methodological biases limited our acoustic detections. Additional research using more sophisticated equipment for recording bat vocalizations, while controlling for confounding factors, could differentiate true vocal silence from obvious detection biases. It remains unknown whether vocal silence is limited to hoary bats and the few times and situations where we observed it, or if silence behavior is more prevalent in this and other species, seasons, and habitat types.

There are practical reasons for developing a better understanding of bat vocal behaviors. For example, acoustic monitoring is a recommended component of preconstruction surveys at proposed wind power facilities (Strickland et al., 2011), with the typical metric being the number of bat passes detected per survey night over an extended survey period. However, the association between acoustic detection and fatality rate appears highly variable among studies, and the evidence for this relationship remains equivocal (e.g., Hein, Gruver, & Arnett, 2013) and may not be reliable for the species dying most frequently at wind turbines (Lintott, Richardson, Hosken, Fensome, & Mathews, 2016). On the other hand, the application of acoustic sampling for other assessments, such as habitat use, species distribution, and trends in occupancy, may be effective given an adequate detection rate relative to sampling duration. For example, occupancy analysis involving a bat species commonly entails a series of nightly acoustic samples, and detection probability simply reflects the proportion of nights with at least one positive detection (Gorresen, Miles, Todd, Bonacorrso, & Weller, 2008). Where species occurrence or activity is relatively high, sampling part or all of a night over multiple nights and sites can produce reliable estimates (e.g., Rodhouse, Vierling, & Irvine, 2011; Weller, 2008), particularly when detectability is partitioned into components attributable to availability (the probability that an animal is available for detection; for example, a bat is present and vocalizes) and perceptibility (the probability of observing the animal, given that it is available for detection; for example, environmental conditions are suitable for acoustic sampling; Reidy, Thompson, Amundson, & O'Donnell, 2016). However, where low abundance or irregular occurrence largely determines the availability of individuals for sampling, silent flight behavior will further contribute to low cue‐production rates. In this situation, acoustic methods may severely underestimate occurrence because although present sometime during a night, bats may not be truly exposed to sampling, thereby resulting in biased and imprecise occupancy estimation (Mckann, Gray, & Thogmartin, 2013). Acoustic crypsis and sampling periods of insufficient duration could jointly contribute to low bat detectability, potentially limiting the effectiveness of acoustic monitoring for many applications (e.g., characterizing habitat use by sparsely distributed species; measuring nightly trends in bat activity; or curtailing wind turbines after detecting bat calls sampled over 10‐min periods).

The practical implications of our findings are not hypothetical or trivial. Unexpectedly high numbers of carcasses of Hawaiian hoary bats have been found beneath wind turbines at multiple wind energy facilities, complicating renewable energy production and conservation efforts for the sole bat species resident in the Hawaiian Island archipelago (Mykleseth, 2017). Acoustic detectors like those we used in this study are commonly used to survey for the occurrence of *L. c. semotus* in natural habits, as well as for applications such as predicting risk before and after turbine construction at wind facilities. On the mainland, fatalities of *L. c. cinereus* compose about 40% of all bats reported at wind turbines, the majority of which are found from late summer through autumn (Arnett & Baerwald, 2013). A similar seasonal pattern has not become apparent in Hawai'i, but the season when turbines pose the greatest risk to hoary bats may also be a time of year when hoary bats are least likely to be vocalizing. Our results raise the uncomfortable possibility that acoustic detection may not be a reliable means of accurately detecting or characterizing activity of Hawaiian hoary bats throughout or at least during certain times of year. Given the current trend toward reliance on acoustic detection as part of conservation and management actions directed toward *L. c. cinereus*, increased understanding of its vocalization behavior clearly is needed.

Despite the difficulties of recording ultrasonic calls of bats flying under natural conditions in the darkness, methods exist for looking further into the possibility of vocal silence in Hawaiian hoary bats and other species. For the current study, we used off‐the‐shelf equipment that was simple to operate and could be set up and left unattended for long periods of time. We saw great promise in the coupling of outdoor thermal surveillance video cameras with acoustic detectors, yet see much room for improvement. One limitation of our approach was that the single‐sensor perspective did not allow precise estimation of where bats were located in the monitored airspace. Multiple cameras and microphones, combined with existing computer processing techniques, would permit the three‐dimensional positions of bats in space to be tracked from acoustic and video data (e.g., Corcoran & Conner, 2016; Seibert et al., 2013). Greater precision of acoustic and video detections could help discriminate recording bias from actual silence. Another promising approach might be use of bat‐mounted echolocation monitoring devices, which could be designed to record or transmit information about the consistency of bat vocalization (e.g., Cvikel et al., 2015). We also encourage investigation into potential sampling biases for a variety of different species of echolocating bats because there could be vulnerabilities to current methodologies that, ironically, limit our ability to understand why bats might stop vocalizing for biological reasons.

## CONFLICT OF INTEREST

None declared.
