# Supplementary material for: Do you hear what I see? Vocalization relative to visual detection rates of Hawaiian hoary bats (Lasiurus cinereus semotus)
Source: Ecol Evol. 2017 Jul 20;7(17):6669–79. doi: 10.1002/ece3.3196 (PMC5587485; doi:10.1002/ece3.3196)
Supplement: Supplementary file 1 [file ECE3-7-6669-s001.docx]

Supplementary Table 1.

Weather data for the Hilo International Airport (12 kilometers north-northeast of the study area) between September 8 and October 14, 2014 obtained from:

www.wunderground.com/history/airport/PHTO/2014/9/16/MonthlyHistory.html?req_city=Hilo&req_state=HI&req_statename=Hawaii&reqdb.zip=96720&reqdb.magic=1&reqdb.wmo=99999

www.wunderground.com/history/airport/PHTO/2014/10/16/MonthlyHistory.html?req_city=Hilo&req_state=HI&req_statename=Hawaii&reqdb.zip=96720&reqdb.magic=1&reqdb.wmo=99999

| Date | Moonlight Phase | Max Humidity % | Mean Humidity % | Min Humidity % | Max Temperature Celcius | Mean Temperature Celcius | Min Temperature Celcius | Max Dew Point Celcius | Mean Dew Point Celcius | Min Dew Point Celcius |
| --- | --- | --- | --- | --- | --- | --- | --- | --- | --- | --- |
| 9/8/2014 | 0.99 | 93 | 76 | 59 | 30.0 | 26.7 | 22.8 | 22.2 | 21.1 | 18.3 |
| 9/9/2014 | 1.00 | 84 | 70 | 55 | 30.6 | 26.1 | 21.1 | 21.1 | 19.4 | 16.7 |
| 9/10/2014 | 0.97 | 84 | 71 | 58 | 29.4 | 25.6 | 21.1 | 21.1 | 19.4 | 16.7 |
| 9/11/2014 | 0.92 | 84 | 67 | 50 | 31.1 | 26.1 | 21.1 | 20.6 | 19.4 | 15.0 |
| 9/12/2014 | 0.85 | 79 | 67 | 54 | 31.1 | 26.1 | 20.6 | 21.7 | 18.3 | 13.9 |
| 9/15/2014 | 0.57 | 82 | 68 | 54 | 31.1 | 26.7 | 21.7 | 22.8 | 20.0 | 16.1 |
| 9/16/2014 | 0.47 | 84 | 72 | 59 | 31.1 | 26.7 | 22.2 | 22.2 | 20.6 | 17.8 |
| 9/17/2014 | 0.37 | 82 | 69 | 55 | 31.7 | 26.7 | 21.1 | 21.7 | 19.4 | 16.1 |
| 9/18/2014 | 0.28 | 87 | 71 | 55 | 31.7 | 26.7 | 21.1 | 22.8 | 20.0 | 15.6 |
| 9/19/2014 | 0.20 | 87 | 73 | 59 | 32.2 | 27.2 | 21.7 | 22.8 | 21.1 | 18.3 |
| 9/22/2014 | 0.03 | 88 | 70 | 51 | 31.1 | 26.7 | 22.2 | 23.3 | 21.1 | 18.9 |
| 9/23/2014 | 0.01 | 90 | 70 | 50 | 31.1 | 26.1 | 21.1 | 22.2 | 20.0 | 15.0 |
| 9/24/2014 | 0.00 | 82 | 66 | 50 | 31.7 | 26.7 | 21.1 | 22.2 | 18.9 | 13.3 |
| 9/25/2014 | 0.01 | 84 | 67 | 50 | 32.2 | 27.8 | 22.8 | 22.2 | 20.6 | 17.8 |
| 10/6/2014 | 0.94 | 90 | 74 | 58 | 31.1 | 26.1 | 21.1 | 22.2 | 20.0 | 16.1 |
| 10/7/2014 | 0.98 | 87 | 69 | 51 | 31.1 | 26.7 | 21.7 | 21.7 | 20.0 | 16.7 |
| 10/8/2014 | 1.00 | 87 | 78 | 69 | 28.3 | 25.0 | 21.1 | 23.9 | 21.7 | 16.7 |
| 10/9/2014 | 0.99 | 94 | 76 | 57 | 30.0 | 26.7 | 22.8 | 22.8 | 21.7 | 20.0 |
| 10/13/2014 | 0.73 | 100 | 81 | 61 | 28.9 | 25.0 | 20.6 | 22.2 | 20.0 | 15.6 |
| 10/14/2014 | 0.63 | 93 | 77 | 61 | 30.0 | 25.6 | 20.6 | 22.2 | 20.6 | 16.7 |

Supplementary Table 1 (continued).

| Date | Max Sea Level Pressure MM | Mean Sea Level Pressure MM | Min Sea Level Pressure MM | Mean Visibility KM | Max Wind Speed KMPH | Mean Wind Speed KMPH | Max Gust Speed KMPH | Precipitation MM | Wind Direction Degrees |
| --- | --- | --- | --- | --- | --- | --- | --- | --- | --- |
| 9/8/2014 | 762.8 | 761.5 | 760.5 | 16.1 | 22.5 | 9.7 | 33.8 | 0.8 | 245 |
| 9/9/2014 | 763.0 | 762.0 | 761.0 | 16.1 | 24.1 | 9.7 | 30.6 | 0.0 | 190 |
| 9/10/2014 | 763.0 | 762.0 | 760.7 | 16.1 | 20.9 | 8.0 | 25.7 | 0.0 | 197 |
| 9/11/2014 | 762.5 | 761.7 | 760.2 | 16.1 | 24.1 | 9.7 | 32.2 | 0.0 | 185 |
| 9/12/2014 | 764.8 | 761.0 | 759.2 | 16.1 | 20.9 | 11.3 | 29.0 | 0.0 | 298 |
| 9/15/2014 | 761.0 | 760.0 | 758.7 | 16.1 | 25.7 | 11.3 | 32.2 | 0.0 | 312 |
| 9/16/2014 | 761.2 | 760.5 | 759.2 | 16.1 | 20.9 | 9.7 | 27.4 | 0.0 | 323 |
| 9/17/2014 | 761.5 | 760.5 | 759.5 | 16.1 | 20.9 | 9.7 | 29.0 | 0.0 | 272 |
| 9/18/2014 | 761.2 | 760.2 | 759.0 | 16.1 | 20.9 | 11.3 | 29.0 | 1.0 | 226 |
| 9/19/2014 | 761.0 | 760.2 | 758.7 | 16.1 | 27.4 | 11.3 | 37.0 | 0.0 | 197 |
| 9/22/2014 | 764.0 | 762.8 | 761.7 | 16.1 | 24.1 | 11.3 | 35.4 | 0.0 | 164 |
| 9/23/2014 | 764.0 | 763.0 | 761.7 | 16.1 | 22.5 | 9.7 | 30.6 | 3.3 | 153 |
| 9/24/2014 | 763.8 | 762.8 | 761.5 | 16.1 | 22.5 | 11.3 | 32.2 | 0.0 | 245 |
| 9/25/2014 | 763.3 | 762.3 | 761.0 | 16.1 | 22.5 | 9.7 | 30.6 | 0.0 | 226 |
| 10/6/2014 | 761.0 | 759.5 | 758.2 | 16.1 | 29.0 | 12.9 | 37.0 | 0.0 | 174 |
| 10/7/2014 | 762.8 | 761.5 | 760.0 | 16.1 | 20.9 | 11.3 | 29.0 | 0.0 | 190 |
| 10/8/2014 | 763.5 | 762.3 | 761.2 | 14.5 | 22.5 | 9.7 | 29.0 | 1.8 | 237 |
| 10/9/2014 | 763.3 | 762.3 | 761.0 | 16.1 | 22.5 | 9.7 | 27.4 | 1.8 | 288 |
| 10/13/2014 | 762.3 | 761.5 | 760.2 | 12.9 | 45.1 | 11.3 | 53.1 | 10.4 | 273 |
| 10/14/2014 | 762.3 | 761.2 | 759.7 | 14.5 | 25.7 | 11.3 | 32.2 | 7.4 | 235 |


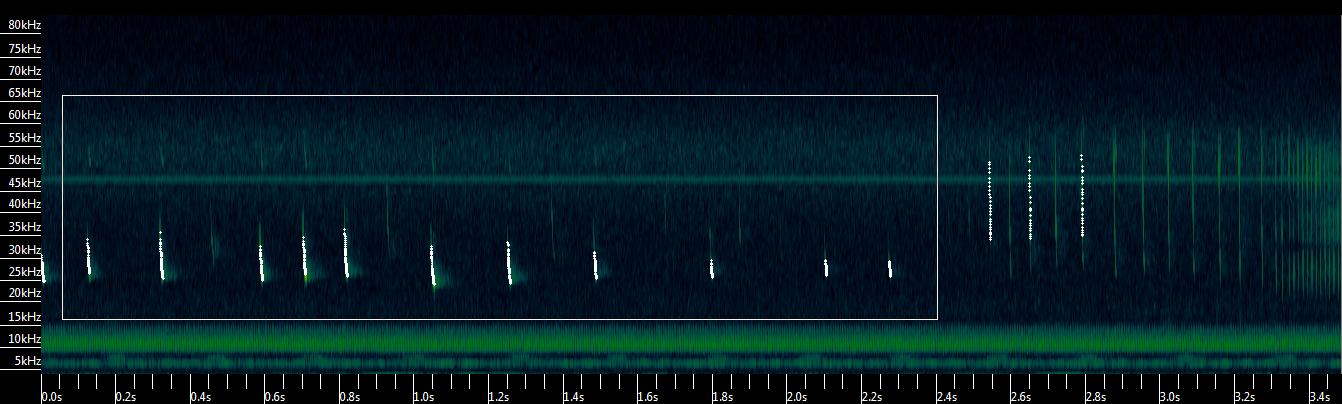


Supplemental Figure 1. Example of the selection (inset box) of the search-phase part of a Hawaiian hoary bat echolocation call used to evaluate structure metrics (note subsequent approach- and terminal-phase portions of the call). Call frequency (kHz) on y-axis and time (second or millisecond) on x-axis of spectrogram. Spectrograms were produced with program Kaleidoscope Pro (version 4.0.0, Wildlife Acoustics, Concord, MA).


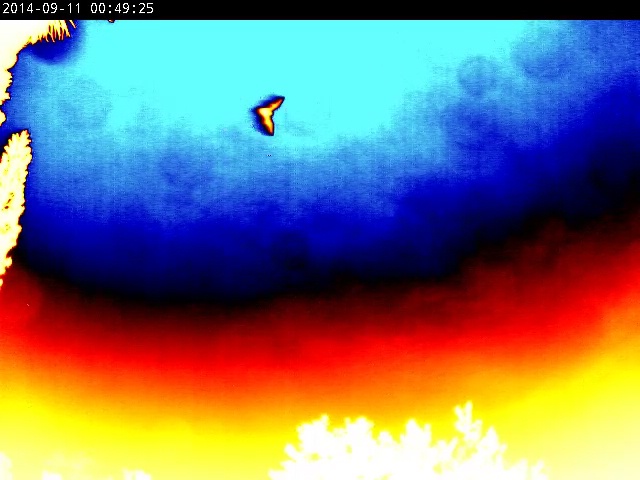


**A**


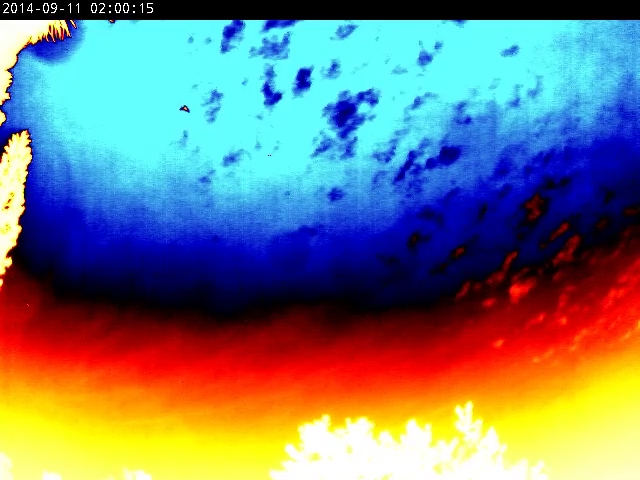


**B**


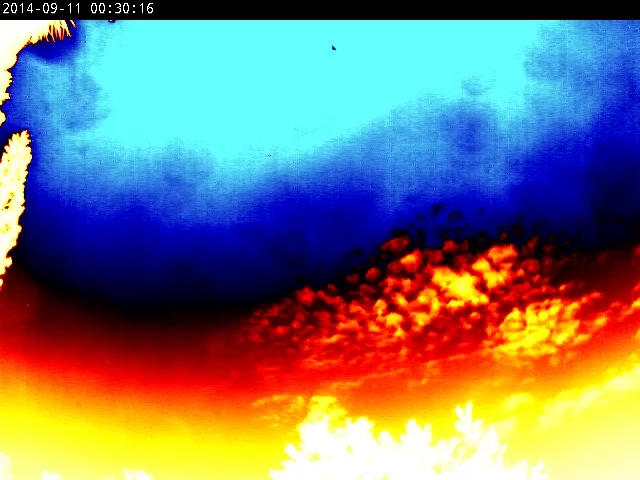


**C**

Supplementary Figure 2. Examples of proximity classes assigned to videographic detections based on the nearest approach by a bat to the camera: (panel A) near-range (≤25 m); (panel B) mid-range (>25 to 50 m); (panel C) far-range (>50 m). Bats detected at a distance of ≤ 50 m were identified by their characteristic body shape and flight, whereas targets > 50 m away were generally identified by flight behavior alone.


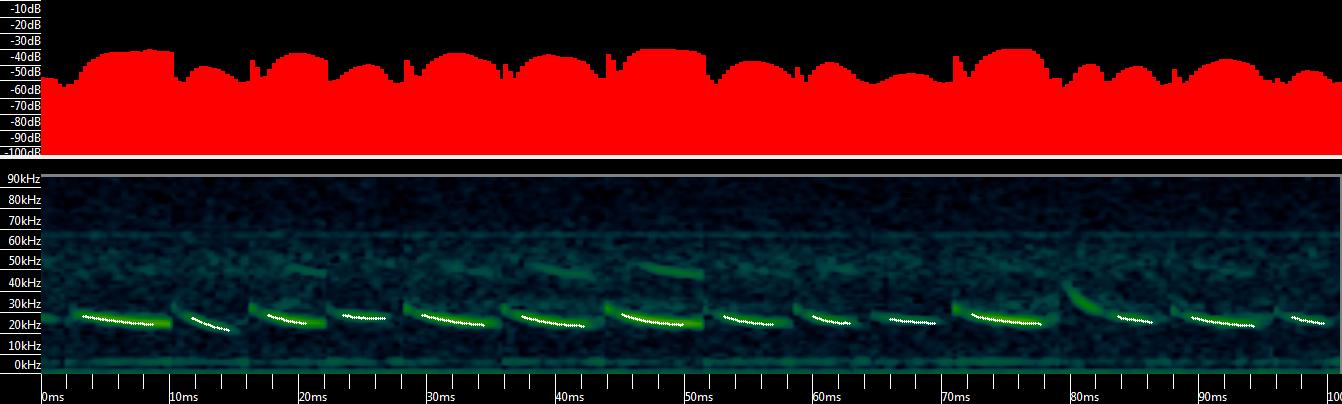


**A**


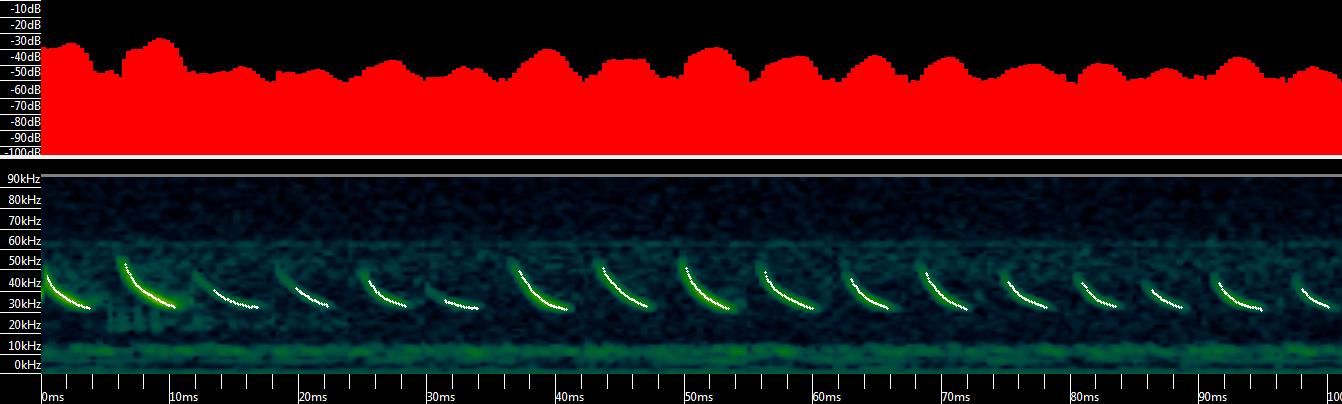


**B**

Supplemental Figure 3. Spectrograms of separate call files with components typical of (panel A) long-shallow narrowband and (panel B) short-steep broadband search-phase calls. The former are used for open-space foraging and are optimized for the long-range detection of weak echoes from insect prey and applied predominantly towards prey acquisition rather than for spatial orientation, whereas the latter are used for edge-space foraging to find prey near acoustic background clutter and to determine position in space in relation to the background (Schnitzler et al. 2003). Note presence of harmonics for some pulses. These spectrograms of a call playback files are presented in a compressed view, leaving out periods of silence between pulses.


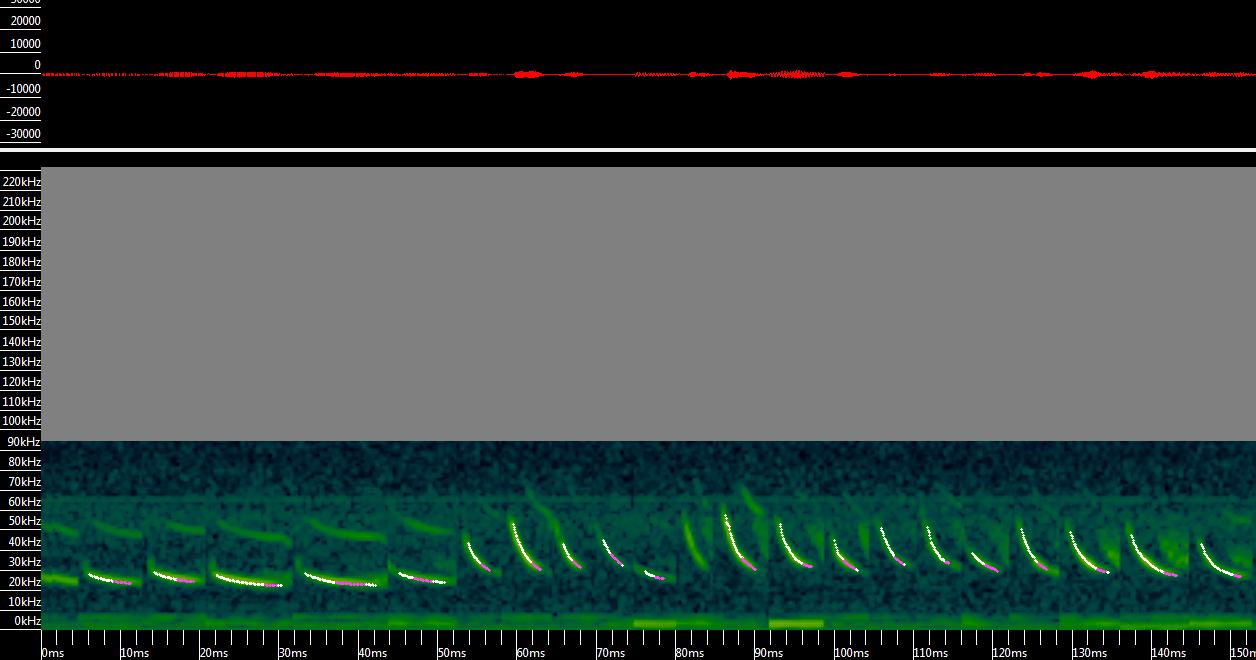


**A**

**B**

Supplemental Figure 4. Spectrogram of a single call file with components comprised of both (panel A) long-shallow narrowband and (panel B) short-steep broadband search-phase calls. This spectrogram of a call playback file is presented in a compressed view, leaving out periods of silence between pulses.


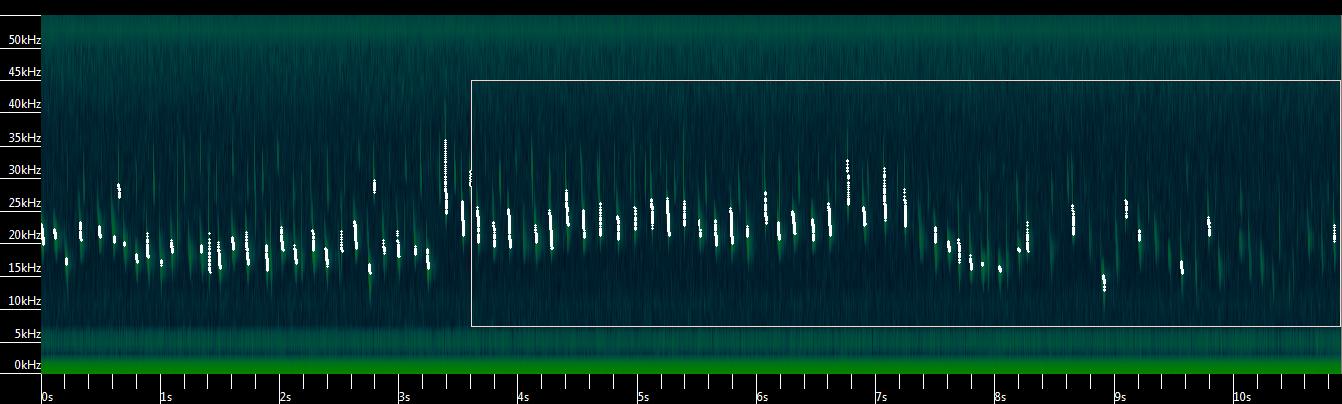


**A**


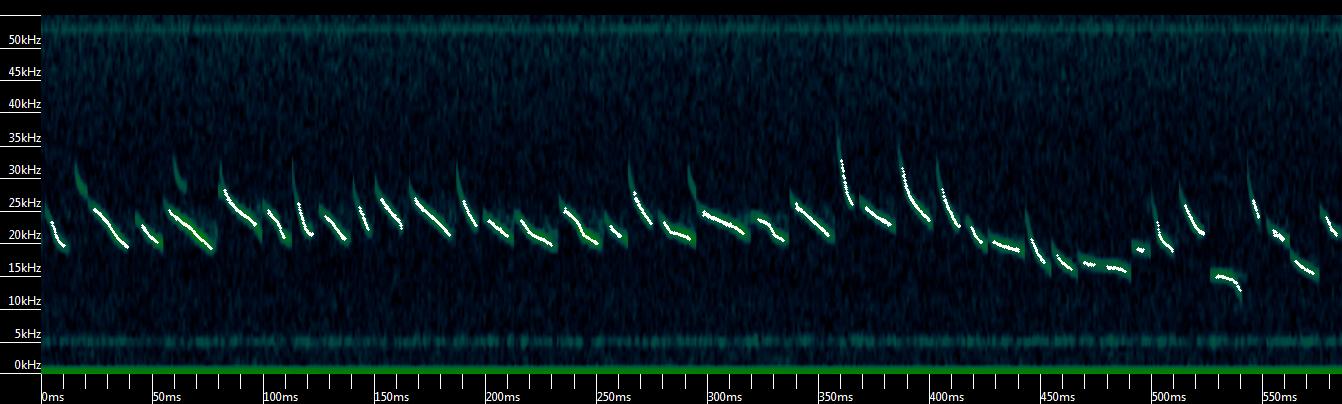


**B**

Supplemental Figure 5. Spectrograms (uncompressed [panel A], compressed [panel B]) involving a pair of bats engaged in a chase (aerial “dogfight”) as determined by synchronized acoustic and visual (videographic) recordings. Box in panel A shows the portion of call depicted in panel B.


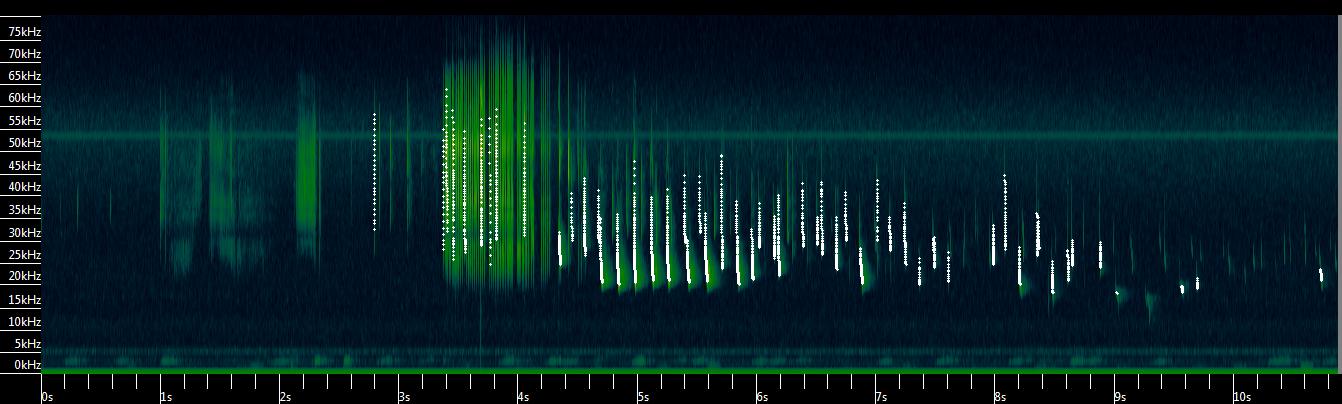


**A**


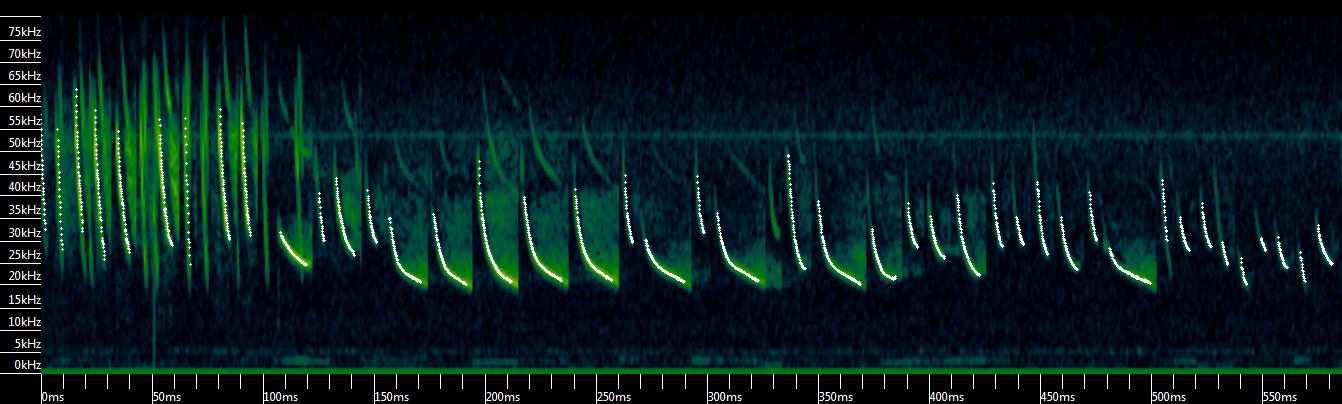


**B**

Supplemental Figure 6. Spectrograms (uncompressed [panel A], compressed [panel B]) involving a pair of bats foraging in close proximity but not engaged in a chase as determined by synchronized acoustic and visual (videographic) recordings.
